# A genomic scale map of genetic diversity in *Trypanosoma cruzi*

**DOI:** 10.1186/1471-2164-13-736

**Published:** 2012-12-27

**Authors:** Raul O Cosentino, Daniel O Sánchez, Fernán Agüero

**Affiliations:** 1 Instituto de Investigaciones Biotecnológicas - Instituto Tecnológico de Chascomús (IIB-INTECH), Universidad Nacional de San Martín - Consejo de Investigaciones Científicas y Técnicas (UNSAM-CONICET), Sede San Martín, B 1650 HMP, San Martín, Buenos Aires, Argentina

## Abstract

**Background:**

*Trypanosoma cruzi*, the causal agent of Chagas Disease, affects more than 16 million people in Latin America. The clinical outcome of the disease results from a complex interplay between environmental factors and the genetic background of both the human host and the parasite. However, knowledge of the genetic diversity of the parasite, is currently limited to a number of highly studied *loci*. The availability of a number of genomes from different evolutionary lineages of *T. cruzi* provides an unprecedented opportunity to look at the genetic diversity of the parasite at a genomic scale.

**Results:**

Using a bioinformatic strategy, we have clustered *T. cruzi* sequence data available in the public domain and obtained multiple sequence alignments in which one or two alleles from the reference CL-Brener were included. These data covers 4 major evolutionary lineages (DTUs): TcI, TcII, TcIII, and the hybrid TcVI. Using these set of alignments we have identified 288,957 high quality single nucleotide polymorphisms and 1,480 indels. In a reduced re-sequencing study we were able to validate ~ 97% of high-quality SNPs identified in 47 loci. Analysis of how these changes affect encoded protein products showed a 0.77 ratio of synonymous to non-synonymous changes in the *T. cruzi* genome. We observed 113 changes that introduce or remove a stop codon, some causing significant functional changes, and a number of tri-allelic and tetra-allelic SNPs that could be exploited in strain typing assays. Based on an analysis of the observed nucleotide diversity we show that the *T. cruzi* genome contains a core set of genes that are under apparent purifying selection. Interestingly, orthologs of known druggable targets show statistically significant lower nucleotide diversity values.

**Conclusions:**

This study provides the first look at the genetic diversity of *T. cruzi* at a genomic scale. The analysis covers an estimated ~ 60% of the genetic diversity present in the population, providing an essential resource for future studies on the development of new drugs and diagnostics, for Chagas Disease. These data is available through the TcSNP database (http://snps.tcruzi.org).

## Background

*Trypanosoma cruzi* is a protozoan parasite of the order Kinetoplastida, and the causative agent of Chagas Disease, one of the so called neglected diseases that disproportionately affect the poor. The disease is endemic in most Latin American countries, affecting in excess of 8 million people [1]. Chagas disease has a variable clinical outcome. In its acute form it can lead to death (mostly in infants), while in its chronic form, it is a debilitating disease producing different associated pathologies: mega-colon, mega-esophagus and cardiomyopathy, among others. These different clinical outcomes are the result of a complex interplay between environmental factors, the host genetic background and the genetic diversity present in the parasite population. As a result, these different clinical manifestations have been suggested to be, at least in part, due to the genetic diversity of *T. cruzi*[2-5].

The *T. cruzi* species has a structured population, with a predominantly clonal mode of reproduction [6], and a considerable phenotypic diversity [7-10]. Through the use of a number of molecular markers the population has been divided in a number of evolutionary lineages, also called discrete typing units. Some markers allow the distinction of two or three major lineages [11-14], while other experimental strategies, such as RAPD and multilocus isoenzyme electrophoresis (MLEE) support the distinction of six subdivisions [15-17] originally designated as DTUs I, IIa, IIb, IIc, IId, and IIe [16]. Recently, this nomenclature was revised as follows: TcI, TcII (former TcIIb), TcIII (IIc), TcIV (TcIIa), TcV (TcIId) and TcVI (TcIIe) [18,19]. Lineages TcV and TcVI (which include the strain used for the first genomic sequence of *T. cruzi*, CL Brener) have a very high degree of heterozygosity but otherwise very homogeneous population structures with low intralineage diversity [20,21]. The currently favoured hypothesis suggests that these two lineages originated after either one or two independent hybridization events between strains of DTUs TcII and TcIII [21-23].

Knowledge of the genetic variation present in a genome (i.e. between the two alleles of a diploid individual) or in a species (i.e. in the population) is of central importance for a variety of reasons and applications: i) to understand the evolutionary forces underlying the biological and phenotypic properties observed in an individual; ii) to detect cases of apparent horizontal gene transfer; iii) to assess the potential for development of resistance when validating a target for drug development; iv) to prioritize targets for development of diagnostics or vaccines; v) in the design of constructs for genetic knockout experiments in order to increase the success rate when targeting specific alleles; and vi) as genetic markers in association studies or to further probe the population structure.

The genome sequence of the CL-Brener clone of *T. cruzi* was published in 2005 [24], together with those of two other trypanosomatids of medical importance: *Trypanosoma brucei* (Sleeping sickness, African trypanosomiasis) [25] and *Leishmania major* (Leishmaniasis) [26]. However, the genome of *T. cruzi* was a particular case for a number of reasons: it was obtained from a hybrid TcVI strain composed of two divergent parental haplotypes; and it was sequenced using a whole genome shotgun strategy [24]. This choice of strain and sequencing strategy resulted in high sequence coverage from the two parental haplotypes, which were derived from ancestral TcII and TcIII strains. Because of the high allelic variation found within this diploid genome, a significant number of contigs were found to be present twice in the assembly [24]. These divergent haplotypes, which were assembled separately in many cases, were the basis of a recent re-assembly of the genome [27]. As a consequence, it is now possible to identify the genetic diversity present within this diploid genome.

More recently a number of whole genome sequencing data have become available from different strains of *T. cruzi*: the draft genomic sequence of the Sylvio X10 (TcI) strain [28], high-coverage transcriptomic data, from another TcI strain (Westergaard G, and Vazquez MP, manuscript in preparation), as well as 2.5X WGS shotgun data from the Esmeraldo cl3 (TcII) strain.

To take advantage of the hybrid genome of the CL-Brener strain, and of other genome and transcriptome datasets, we designed a bionformatics strategy to obtain information on the genetic diversity present in these data. As already observed for a significant number of molecular markers, each of the alleles identified in the majority of the polymorphic heterozygous site in strains from hybrid lineages TcV and TCVI can be observed in homozygosity in strains from either of the two proposed parental lineages (TcII and TCIII) [20,21,29-31]. Therefore by uncovering the diversity within the CL-Brener and Sylvio X10 genomes, we expect to reveal a significant fraction of the diversity that can be observed between extant TcI, TcII, TcIII, and TcVI strains.

In this work we present an initial compilation of a genome-wide map of genetic diversity in *T. cruzi*, and its functional analysis, focussed mostly on protein-coding regions of the genome.

## Results

### Sequence clustering, alignment and identification of polymorphic sites

To identify genetic variation in *T. cruzi* we took advantage of available sequence data in public databanks, including the genome sequence of the CL-Brener and Sylvio X10 strains, expressed sequence-tags and other sequences submitted by independent authors to these databanks. Our strategy to map this diversity relied on the generation of multiple sequence alignments and on the scanning of these alignments to identify polymorphisms [32]. As mentioned, the sequence of the *T. cruzi* genome was obtained using a whole genome shotgun strategy, from a hybrid clone (CL-Brener) [24]. Because of the sequence divergence between alleles of the CL-Brener clone, assembly of this genome resulted in many cases in the separation of these alleles into separate contigs [24,27]. This allowed us to align these sequences and identify sequence differences. However, because of the repetitive nature of the *T. cruzi* genome [24,33], we decided to focus this initial effort on mapping the genetic diversity in mostly single copy protein coding *loci*. These were defined as those sequences represented by no more than 2 coding sequences from the CL-Brener (reference) genome in our sequence alignments (see below).

Sequences used in this work include all the annotated coding sequences from the reference CL-Brener genome, and the corresponding coding sequences (CDS) from the Sylvio X10 genome, as well as other publicly available sequence data (see Table 1). After clustering sequences by similarity (see Methods) we obtained 7,639 multiple sequence alignments, 71.3% of which had 2 reference coding sequences from the CL-Brener genome (and therefore most probably representing single copy *loci*; see Table 1). Other alignments contain increasing numbers of reference coding sequences. These set of alignments contains sequences for most of the large gene families of *T. cruzi*, and were not considered further. Even after this stringent filtering, there were still a number of alignments that contained only two reference sequences from the CL-Brener genome, but that belonged to these large gene families – mucins, mucin-associated proteins (MASP), *trans*-sialidase-like proteins, etc. These correspond to cases where highly similar copies of members of a family were separated from their paralogs during the clustering or assembly steps. Finally, a number of alignments (~6%) had only one reference sequence from the CL-Brener hybrid. These cases may correspond to haploid regions in the hybrid genome or to cases where two highly divergent alleles were separated during the clustering step.

**Table 1:** Sequences, alignments and SNPs: summary of data generated and analyzed in this work

**Description**	**Number**
**Sequences**	
CL-Brener Reference (CDS); TcVI	25,013
Mapped CDS from Sylvio X10 genome; TcI	4,918
Mapped transcripts from TcI transcriptome	3,486
Mapped reads from Esmeraldo cl3 shotgun; TcII §	134,424
Mapped Expressed Sequence Tags (ESTs)	13,968
Mapped misc GenBank sequences (mRNAs, CDS)	2,038
**Alignments**	
Total	7,639
Containing two reference coding sequences	5,447
**SNPs**	
Total	325,355
With P > 0.70 †	305,993
In good sequence neighborhood *	302,390
P > 0.70 AND good seq neighborhood †*	288,957
Synonymous †*	125,455
Non-synonymous †*	162,820
Nonsense †*	113
Non-coding †*	569
Triallelic †*	2,990
Tetra-allelic †*	10
Average SNP density †*	2.4 per 100 bp
**Indels**	
Total	28,316
With P > 0.70 †	11,007
In good sequence neighborhood *	10,523
P > 0.70 AND good seq neighborhood †*	1,480

§ reads where at least 50bp matched the reference with >= 95% identity.

† SNPs with probability > 0.7 as assigned by PolyBayes [34].

* SNP is located in a 10 bp window with < 2 other SNPs.

We then scanned the multiple sequence alignments and identified columns containing sequence differences and/or indels. From the set of all alignments we identified 325,355 sites with variation (putative single nucleotide polymorphisms, or fixed differences), of which 28,316 corresponded to small indels (Table 1). These polymorphic sites provide representative information on the diversity found in *T. cruzi* evolutionary lineages TcI (Sylvio X10), TcVI (CL-Brener), but also in lineages TcII and TcIII (represented by the variation found within the CL-Brener hybrid).

Columns containing variation in a multiple sequence alignment may correspond to polymorphic sites (SNPs) or to sequencing errors. To discriminate between these possibilities, we also analyzed the sequence neighborhood around each potential SNP. Based on this analysis we found 302,390 SNPs (and 10,523 small indels) located in regions with a low density of SNPs (good sequence neighborhoods, with < 3 SNPs in a 10 bp window, see Methods). To further assess the quality of the sequence around/in each SNP we used a statistical software package (PolyBayes [34]) together with quality values for each base that were derived from the expected error rate for each sequence (described in Methods). Using this approach we identified 288,957 SNPs (and 1,480 small indels) that have both a high probability according to PolyBayes (p > 0.7) and are located in good sequence neighborhoods. Using this conservative set of SNPs, we obtained a density of 2.4 SNPs per 100 bp for *T. cruzi* coding regions.

The great majority (99%) of the observed SNPs were bi-allelic (changing one specific nucleotide base for another), however there were 2,990 tri-allelic SNPs (0.98%) and 10 tetra-allelic SNPs (0.0033%, P > 0.7). These are very interesting SNPs that can be exploited in the design of strain typing assays. One such assay, based on one tetra-allelic and a number of tri-allelic SNPs has just been developed using this information [31].

All this information is available in the Additional file 1: Table S1 and has also been integrated in a new release of the TcSNP database [32].

#### Experimental validation of candidate SNPs

To validate the strategy used *in silico*, and to assess the quality of the SNPs and the probability of them being true SNPs (as opposed to sequencing errors) we performed a small scale re-sequencing study on 47 *loci* (see Table 2). This set contained 1136 predicted SNPs with probabilities (as reported by PolyBayes) ranging from 0 to 1, obtained from genes with different numbers of predicted polymorphisms: low (e.g. 1–10 predicted SNPs), medium (11–25 SNPs) and high (> 25 SNPs). PCR amplification of selected fragments from these *loci* was followed by direct sequencing of the amplified products and identification of SNPs from the raw chromatogram sequence data, including heterozygous peaks (using Polyphred for this [35]). This re-sequencing experiment allowed us to validate ~ 96% of the predicted SNPs that had PolyBayes probabilities >= 0.7 (Table 2), whereas the success rate for SNPs with probabilities between 0–0.4 fell to 12.5%. The results of this small-scale study suggest that overall the scoring strategy used to rank the SNPs worked well. We also identified 43 new heterozygous SNPs within the CL Brener strain (SNPs not predicted by our *in silico* approach and not present in the original release of the TcSNP database [32]) and 1,261 new SNPs from other *T. cruzi* strains (RO Cosentino, L Panunzi, F Agüero, unpublished). The majority of these new CL-Brener SNPs escaped the initial *in silico* prediction because of artifacts in the assembly of the *T. cruzi* genome, which resulted, for example, in a missing allele for an hypothetical protein (TcCLB.511003.60) with high similarity to the yeast ERG10 gene (Acetyl-CoA C-acetyltransferase). In the *T. cruzi* genome database there is only one allele reported for this gene. As a consequence, the few polymorphisms identified by our computational strategy were derived from the comparison of this allele against a short CL-Brener EST sequence (TENG0250, accession number 3258889, see alignment tcsnp:436105). However upon PCR amplification from CL-Brener DNA, we were able to uncover additional heterozygous polymorphisms (see GenBank accession numbers HQ586976, and HQ586977). Both pieces of evidence suggest that there is a second allele that was probably merged or missed during genome assembly. Apart from this case, this small scale re-sequencing experiment confirmed the majority of the SNPs identified *in silico*, which is in agreement with the expected sequence coverage/quality of genomic and transcriptomic data used. A complete table listing all *loci* analyzed, and their SNPs is available in Additional file 2: Table S2.

**Table 2:** Validation of the SNP scoring scheme by PCR-based re-sequencing

	**SNP score range**						**Total**	
	**0–0.4**		**0.4–0.7**		**0.7–1.0**		**(0–1)**	
	**V**	**NV**	**V**	**NV**	**V**	**NV**	**V**	**NV**
No. of SNPs	9	63	25	8	996	35	1030	106
Percent validation	12.5		75.76		96.61		90.67	

Fragments from selected *loci* were amplified by PCR and directly sequenced, and SNPs were identified using PolyPhred after base-calling. The table shows the number of SNPs identified *in silico* that have been validated (V) or not validated (NV), grouped by their score.

Based on the results from this re-sequencing experiment we decided to focus our analysis of genetic diversity on the subset of high-quality SNPs (P > 0.7) that are also located in regions of good sequence neighborhood. This subset was therefore used throughout the study. Because the candidate allelic copies of each reference coding sequence are now aligned in our dataset, we use the words *gene* and *alignment* interchangeably to refer to the genomic *loci* represented by these sequences.

#### A first genome-wide look at the genetic diversity of *T. cruzi*

In the subset of high-quality SNPs, we first looked at the types of changes observed at the DNA level: transitions and transversions. Theoretically, there are twice the number of possible transversions than transitions. However, because of the nature of the molecular mechanisms involved in the generation of these mutations transitions are found more frequently than transversions [36,37]. And *T. cruzi* was not exception. As observed previously for rRNA genes [38] we observed an excess of transitions (70.1%) over transversions (29.1%, see Table 3).

**Table 3:** Transitions and transversions in T. cruzi

		**Observed counts**				
**Substitutions**		**Global (all)**	**Synonymous**	**Non-synonymous**	**Non-coding**	**Nonsense**
transitions (ts)	AG + GA	104,421	45,652	58,581	170	18
	CT + TC	96,208	55,758	40,200	208	42
transversions (tv)	AC + CA	29,973	7,739	22,168	48	18
	AT + TA	13,537	4,229	9,266	31	11
	CG + GC	17,461	4,110	13,284	64	3
	GT + TG	27,357	7,967	19,321	48	21
**Ratio ts / tv**		2.27	4.22	1.54	1.98	1.13

The set of high-quality SNPs was used to count the ocurrences of transitions and transversions in different relevant partitions of the data.

When analyzing the subset of high-quality SNPs at the codon level, SNPs were more frequently observed at the 3rd codon position (53%), followed by the 1st codon position (25%) and the 2nd (21%).

#### Functional characterization of polymorphic sites: nonsense SNPs

Using the set of high quality SNPs (p > 0.7, in good sequence neighborhoods) we observed 76,452 silent (synonymous) SNPs, 99,552 non-synonymous SNPs and 161 non-sense SNPs – those introducing or removing stop codons in proteins (see Table 1).

After manual inspection of alignments containing nonsense SNPs, to filter out cases that could be explained by genome assembly problems, we ended up with 113 alignments (*loci*) with clear nonsense polymorphisms, many of which correspond to hypothetical proteins (80%, see Additional file 3: Table S3). These nonsense polymorphisms were produced by changes affecting different positions of the codon (see Additional file 4: Figure S1).

Interestingly, we also observed a bias in the codon position affected by these nonsense SNPs. Even though, theoretically, we would expect nonsense SNPs in the 1st base of a codon in 9 out of 23 nonsense SNPs (39%), we observed a significantly higher number of nonsense SNPs arising from mutation of the 1st base of a codon (66/110, 60%, p < 0.0005 Chi-square test for the comparison of the observed-expected values for all 3 positions.

To further inspect whether these changes have been fixed by evolution or were potentially recent events, we analyzed these 113 *loci* in additional strains: the JR cl4 and Esmeraldo cl3 strains, taking advantage of preliminary assemblies available at the TriTrypDB resource [39]. These data is available in Additional file 3: Table S3.

Depending on which allele we consider as the ancestral allele, we can describe these SNPs as either introducing a premature stop codon (therefore producing a truncated protein) or as generating a read-through codon (therefore producing a longer product). The comparison of nonsense mutations in the available data suggest that in 3 cases (hypothetical proteins TcCLB.509767.140, TcCLB.503905.50, and TcCLB.506691.70) the ancestral state of the codon was most probably a STOP (readily identified in 5 out of 6 strains) that was changed into a read-through codon in one strain/lineage only. In other cases the situation might be similar (TcCLB.506773.80, TcCLB.510533.80, TcCLB.508207.170, TcCLB.503525.4, TcCLB.508567.49, TcCLB.505229.20,  all hypothetical proteins;  and TcCLB.508181.140, an inorganic pyrophosphatase), although the corresponding CDS was missing from one of the strains. In contrast, in 44 cases (39%) the nonsense mutation was only observed once, and can therefore correspond to the alternative case, in which the ancestral codon was replaced by a premature stop, therefore generating a truncated protein product.

Analysis of the these cases, revealed that the majority of them (77%) contained the nonsense SNP in the final 10% of the corresponding coding sequence, near the 3’ end of the other allele (see Table S3), and therefore may not be associated with large functional changes. However, in a few cases the identified nonsense SNPs are producing predicted disruptive changes.

In the case of a putative GDP-mannose 4,6 dehydratase (alignment tcsnp:439464, TcCLB.503881.20), the nonsense SNP, present only in strains from the TcI lineage, is located near the N-terminus of the protein, therefore theoretically resulting in a complete truncation. Although there is a downstream ATG that could be used to produce a product with only a 11% reduction of its size, this product would lack the conserved NAD(P) nucleotide binding motif GGxGxxG [40], and therefore we believe it cannot produce a  functional  protein.  In  another  case  (alignment  tcsnp:438249, including reference sequences TcCLB.511907.190 and TcCLB.506801.70), the presence of a nonsense SNP in one CL-Brener allele, causes the shorter TcCLB.506801.70 allele to lose a potential glycosylphosphatidyl inositol C-terminal anchor sequence, generating a potential significant change in localization of the protein (secreted vs membrane anchored product). The number of SNPs identified between these two sequences is approximately twice the average found in other sequences (117 SNPs, 47 non-synonymous SNPs). This, together with the observed differences in sub-cellular targeting signals, suggests that these alleles may have divergent functions. Another case involving a potential change in sub-cellular localization due to a missing GPI-anchor in one allele, was identified in alignment tcsnp:442281 (containing reference sequences TcCLB.508951.80 and TcCLB.510121.80), encoding a putative proteins that belongs to the RNI-like superfamily of leucine-rich containing proteins, which are thought to mediate protein-protein interactions.

### Distribution of SNPs in *T. cruzi* coding regions

Next, we analyzed the distribution of SNPs along the coding region, and in the context of different sequence features: *trans-*membrane domains, signal peptides, globular vs unstructured regions. We reasoned that the selection acting on the gene might be different in these different regions or domains. Based on this idea, we performed a number of comparisons, evaluating differences in the density of synonymous and non-synonymous changes in one of these domains vs the rest of the protein. However, although some significant signal can be observed when performing pairwise comparisons (e.g. between the Esmeraldo-like and Non-Esmeraldo-like alleles of CL-Brener), these differences are not significant when using the complete data that includes alleles from TcI, TcII (Esmeraldo, and Esmeraldo-like from CL-Brener), TcIII (non-Esmeraldo-like alleles from CL-Brener), and TcVI (CL-Brener).

**Figure 1:**
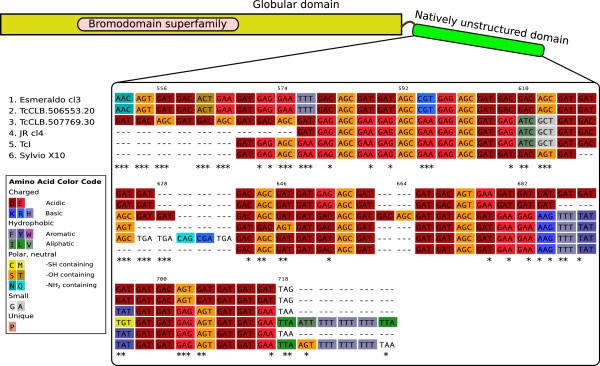
**A natively unstructured domain accumulating non-synonymous changes.** Section of a multiple sequence alignment (starting at position 550 in the alignment) showing the C-terminal, natively unstructured, region of the TcCLB.506553.20 gene, and its allelic counterparts in other strains. Asteriks indicate non-synonymous changes. The nucleotide multiple sequence alignment was obtained with TranslatorX, using the aminoacid translation as a guide [71]. The complete multiple sequence alignment is available as a Additional file 6.

One of the features analyzed, was the presence of SNPs in natively unstructured domains. Several recent papers report an observation that natively unfolded domains can support higher non-synonymous substitution rates [41,42]. Based on predictions made using IUPred [41] we identified globular and natively unstructured domains in *T. cruzi* proteins (globular regions ranged from 10 to 90% of the protein). A comparison of the SNP density found in these regions showed no statistically significant differences (data in Additional file 5: Figure S2). However, we did observe a great dispersion in the density of SNPs in non-globular regions, with more outliers with higher densities of non-synonymous SNPs in this category. Analysis of the functional annotation of these outliers showed enrichment in transporters, kinases (including some 13 protein kinases with no known function) and hydrolases (including a number of ubiquitin hydrolases). A particularly striking outlier is the TcCLB.506553.20 gene encoding a bromodomain containing protein, with a restricted phylogenetic distribution. As can be seen in Figure 1 (C-terminal domain) and in the Additional file 6 (complete alignment), in which we also analyzed the alleles present in preliminary assemblies of the JR cl4 and Esmeraldo cl3 genomes, 70 out of a total of 94 SNPs (87 of which are non-synonymous), were located in a natively unstructured C-terminal tail. Besides being present in all trypanosomatids, this gene is also present in *Trichomonas* and in a a few other organisms such as *Caenorhabditis*, *Cryptosporidium*, and in one plant (*Oryza sativa*).

Another interesting gene showing a striking accumulation of non-synonymous changes in a natively unstructured domain is the A2Rel-like protein of *T. cruzi* (alignment tcsnp:435203, alleles TcCLB.506859.110, TcCLB.511815.80), which was first described in *Leishmania*[43,44]. In this case the majority of SNPs identified are located in a disordered N-terminal domain, as predicted by IUPred.

#### Assessment of selection pressure in *T. Cruzi* coding genes

**Figure 2:**
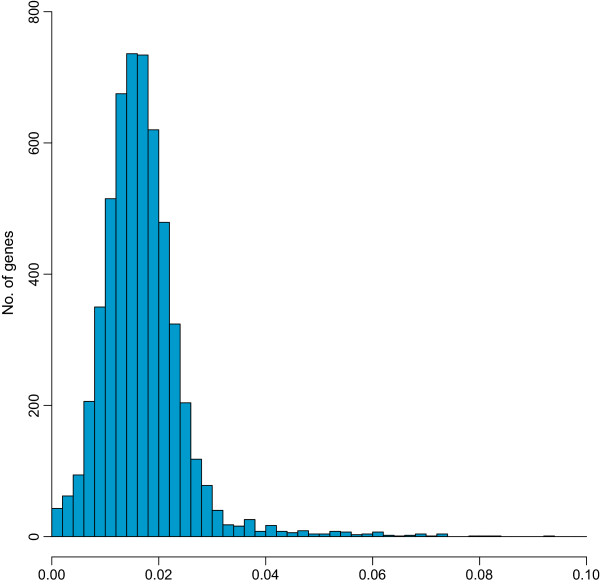
**Allelic divergence observed in T. cruzi.** Histogram of nucleotide diversity (π), showing the distribution of allelic variation in the analyzed data. Alignments (*loci*) meeting our criteria for good quality (see main text) and containing no more than 2 reference coding sequences from the CL-Brener genome, were used to calculate the nucleotide diversity values in the figure. The values of π were normalized over the effective length of the alignment (see Methods). For the histogram, the values were binned as shown in the x axis. Only values of π < 0.1, corresponding to 7,624 alignments (99.8%) were included in the graphic.

Because SNPs identified in this work represent variation observed within a species, we decided to use the nucleotide diversity indicator π as an estimate of selection [45,46]. In our set of high-quality alignments (at most 2 reference coding sequences from the CL-Brener genome), π ranged between 0 and 0.15 (Figure 2).

Not taking into account *loci* corresponding to singleton sequences (those not grouped and aligned with any other sequence), the remaining *loci* with nil values of π were those for which we could not identify high-quality SNPs (for example sequences aligned against 100% identical copies and/or mRNAs). As seen in Figure 2, there is an apparent enrichment of alignments with no SNPs identified. By inspecting the annotation of these genes, it is clear that many of these cases correspond to alignments containing highly identical copies of genes from large families. It has been observed already that many of these genes (e.g. mucins) are organized in tandem arrays, where copies of the array display unusually high nucleotide identity values [47]. It is clear that the diversity observed in one of these alignments (where highly identical sequences from a larger family were clustered and aligned together) is not representative of the overall diversity that can be seen at the family level (e.g. between paralogous copies). Apart from these cases, alignments with low *π* values were those of ribosomal proteins, histones and cytochromes among others.

**Figure 3:**
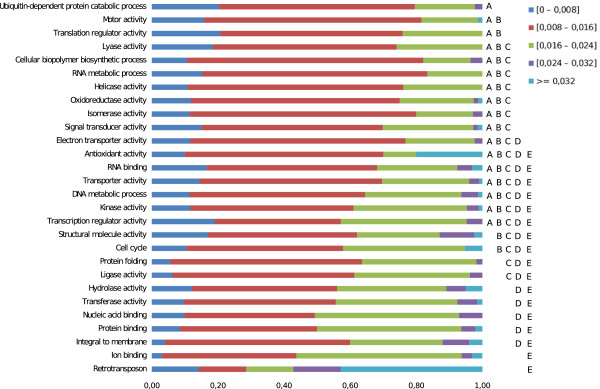
**Genetic diversity in functional classes (Gene Ontology).** Alignments containing genes annotated with GO terms (2,158 alignments) were grouped into higher-level classes based on the GO ontology of terms using GOSlim, and also based on nucleotide diversity ([0–0.008); [0.008–0.016); [0.016–0.024); [0.024–0.032); >= 0.032). Differences between categories were observed and are marked in the figure (Kruskal-Wallis test, P < 0.0001; *a posteriori* test, P < 0.05). Different letters indicate significant differences.

**Figure 4:**
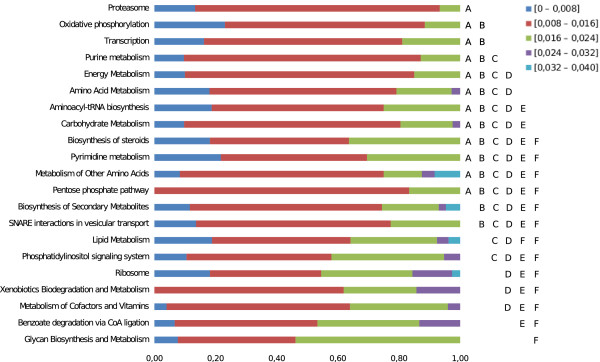
**Genetic diversity in metabolic pathways.** 657 genes with EC numbers were mapped to the corresponding metabolic pathways in the KEGG database [72] and sorted based on the nucleotide diversity (π) estimator of the selection acting on the gene. Different letters (on the right) mean statistically significant differences (Kruskal-Wallis test, P < 0.0013; *a posteriori* test P < 0.05).

To assess the functional relevance of the nucleotide diversity indicator, we looked at the distribution of π in different functional contexts: the functional annotation of the *T. cruzi* genome using the Molecular Function ontology (from the GO Gene Ontology); and the functional mapping of *T. cruzi* enzymes in metabolic pathways according to the KEGG Metabolic Pathways database (data available in Additional file 7: Table S4 and Additional file 8: Table S5). First, using a subset of terms from the Gene Ontology (GO Slim) [48] we grouped 2,158 alignments (*loci*) containing GO annotation into 27 broad classes as defined by their parent GO terms from the Molecular Function ontology. There were significant differences in the π values when comparing all classes using the non-parametric Kruskal-Wallis test (p < 0,0001, *a posteriori* test p < 0.05; see Figure 3). The categories showing less diversity were those with functions in oxidative stress response, protein ubiquitination, and those involved in RNA processing and translation. On the other extreme, classes showing a high nucleotide diversity were those corresponding to integral membrane proteins, ion binding (mostly Ca^++^) and retrotransposons. In all cases, we observed a significant dispersion of the π indicator for any one class. Secondly, we performed a similar analysis on genes with assigned EC numbers (655 alignments) that were mapped onto KEGG pathways. In this case, genes that participate in transcription and protein degradation (proteasome) showed less nucleotide diversity, similar to what we observed for GOSlim classes; whereas genes involved in glycan synthesis and degradation, metabolism of co-factors and vitamins, and xenobiotic metabolism showed a higher nucleotide diversity (Kruskal-Wallis test, p < 0.004, *a posteriori* test p < 0.05; see Figure 4).

The observed dispersion of the apparent selection pressure acting on a given metabolic pathway is not surprising, as the importance of different steps in the pathway (e.g. their centrality, connectivity) is not homogeneous. In the case of *T. cruzi*, the sterol biosynthesis pathway (one of the few validated pathways for drug development) is a nice example of this observation. Interestingly, current validated targets (e.g. the lanosterol demethylase and the C-24 sterol reductase) display low numbers of non-synonymous changes (10% and 28% non-synonymous SNPs, and π = 0.015, and 0.0065, respectively). However, at the same time, other enzymes of the pathway like the C-5 sterol desaturase (alignment tcsnp: 440342) apparently not required by the intracellular amastigotes [49] is accumulating more non-synonymous polymorphisms (53% non-synonymous SNPs, π = 0.012, although still at a level corresponding to a neutral accumulation of changes).

#### Predicted druggable targets display less genetic diversity in *T. cruzi*

Attractive targets for drug development have to meet a number of requirements. The most important of these is the essentiality of the target for survival of the parasite within the host. However, a number of other criteria are often used to prioritize drug targets [50], druggability – knowledge about inhibition or modulation of the target by a small molecule – being one such criteria. For human pathogens, the druggability of targets in whole genomes has been predicted based on their similarity against a database of known druggable targets, and on the presence of a number of sequence, and structural features [51]. Druggability predictions are available from the TDR Targets database [52] in the form of a druggability index associated with each target that goes from 0 (not druggable, or lack of prior information on druggability) to 1 (druggable, highly similar to a target with a known inhibitor/drug). For *T. cruzi* druggability predictions allowed the identification of 173 *loci* with a druggability index > 0.6.

**Figure 5:**
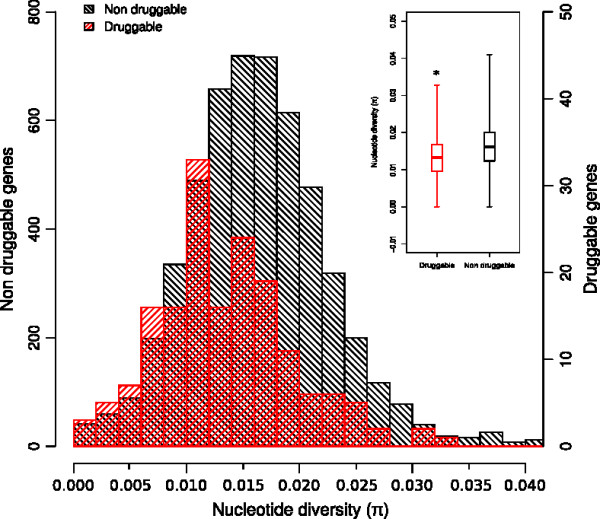
**Genetic diversity of *T. cruzi *druggable genes.** The plot shows the distribution of the nucleotide diversity (π) estimator for *T. cruzi* druggable genes (125 genes, druggability index >= 0.6 [70]), and for genes that are either non-druggable or lack information about their druggability. The two distributions are statistically different according to the Wilcoxon test (P < 0,0001).

In the context of the selection of drug targets for drug discovery, the evolutionary forces acting on a gene may be used as a surrogate marker for essentiality or to assess the risk of development of drug resistance. Taking advantage of the genetic variation identified within the *T. cruzi* genome we analyzed the apparent selection pressure in predicted druggable targets (druggability index >= 0.6) vs the rest of the genome – genes encoding products that are either not druggable (druggability index < 0.6) or for which there are currently no information about their druggability (5,231 genes). For this analysis we used the nucleotide diversity indicator π, or the dN/dS indicator (not shown). We then analyzed the distribution of π in these two groups of genes (see Figure 5). The two distributions were statistically different (Wilcoxon test, P < 0.0001), with the distribution of druggable targets shifted to lower values of π. This showed that a significant number of targets that are predicted to be druggable in *T. cruzi* displayed less variation (i.e. they are under apparent purifying selection). This may not be surprising, as many druggable targets are highly conserved genes with housekeeping cellular functions [53]. However, this result also highlights the fact that there are many genes, with low nucleotide diversity values, for which we don’t have information about their druggability (e.g. overlapping red/gray bars in quartile 1 of the distributions in Figure 5). Targets in this portion of the curve might represent novel classes of targets, not previously explored (e.g. parasite specific targets), and thus worthy of consideration.

Following this reasoning, we suggest that it may be helpful to include genetic diversity information in target prioritization strategies, such as those discussed by Crowther *et al.*[54]. We therefore used the genetic diversity information described in this work to prioritize *T. cruzi* targets (see strategy in Methods), using a genetic diversity score based on a normalized inversion of the nucleotide diversity value. With this scoring system, genes with a low π value will attain a higher score, and *vice versa* (see Methods). The result of this prioritization is available from Additional file 9: Table S6. In this list, three validated targets appear within the top 12 targets: the lanosterol 14-α demethylase (target of triazoles), the trypanothione reductase (essential in all trypanosomatids studied so far), and the protein farnesyltransferase (target of the drug tipifarnib), therefore serving as controls of the prioritization strategy. The list highlights a number of interesting targets, some of which are currently unexplored in *T. cruzi*. The top ranked target in this list is a dihydrolipoamide dehydrogenase (EC 1.8.1.4), an enzyme usually involved in both energy generation and defense mechanisms against oxidative and nitrosative attacks. Selective inhibitors for this enzyme have been identified in *T. cruzi*[55] and *Mycobacterium tuberculosis*[56]. The allelic variants identified display limited variation, with a π value of 0.01, and only 4 non-synonymous SNPs. Other interesting targets displaying low nucleotide diversity values include the RNA polymerase III (the target of tagetitoxin), an S-adenosylhomocysteine hydrolase that displays large biochemical differences with its human orthologue [57], a unique deoxyuridine triphosphatase [58], an inorganic pyrophosphatase, and a number of aminoacyl tRNA synthetases, amongst others. A detailed analysis of this prioritized list is not within the scope of this work and will certainly be done by interested researchers in the field. However, it is worthwile to mention that using genetic information provides an additional, independent, measure to prioritize targets, selecting those that are under apparent purifying selection.

## Discussion

### Identification and ranking of SNPs

In this study we identified hundreds of thousands of candidate SNPs located primarily in single-copy coding regions of the *T. cruzi* genome. The scoring strategy used to evaluate putative SNPs in the absence of raw chromatogram data proved to work reasonably well, as we were able to confirm >= 96% of the SNPs having a probability value >= 0.7 by re-sequencing. The genome-wide data shows that the *T. cruzi* genome is highly polymorphic, with almost 5 polymorphic sites every 200 bp (2.4%). This figure is similar, though not identical, to that reported by El-Sayed *et al.* for the CL-Brener whole genome (2%) [24]. However, there are differences in both strategies. In our case, the analysis is focused mostly on coding regions. This would have resulted in a slightly lower nucleotide diversity value if we had limited our analysis to the CL-Brener hybrid. However, by inclusion of data from other strains, we effectively sampled more genetic space, therefore arriving at a significantly higher diversity for protein coding regions.

The *T. cruzi* genome is highly repetitive [24,33] with many large gene families often organized in tandem arrays. Assembly of this type of genomes from whole DNA shotgun is expected to be difficult [59], and the *T. cruzi* genome was no exception [24]. This could have made the identification of SNPs problematic, or caused the identification of false SNPs (misassembly, and/or sequencing errors). Nonetheless, the high sequence coverage attained (~ 11X for both CL-Brener and Sylvio X10 genomes) resulted in high confidence sequence (at the base level) for most of the single-copy portions of the genome. We have targeted these portions of the genome for mapping polymorphic sites, reasoning that these are the regions with a higher quality. This is certainly not true for all cases, as has been recently noted [60]. However, our strategy for validation of putative SNPs by re-sequencing worked reasonably well. The methodology was based on direct sequencing of PCR amplification products, and except for three cases, we did not observe amplification of bands of different sizes or an excess of heterozygous peaks in the sequence traces, which would be suggestive of amplification of a mixture of genes from different *loci* (e.g. paralogous copies). From a total of 47 *loci* that were selected for re-sequencing, one was discarded due to the presence of amplification products of different sizes, another was the result of an amplification of a tandem array containing two copies of the targeted gene (N-acetylglucosaminyl-phosphatidylinositol-  deacetylase,  putative; TcCLB.511481.40), while in the third case the excess of heterozygous sites revealed the missing allele for a single-copy gene (TcCLB.511003.60, see Results). Except for these cases, all other targeted *loci* showed the expected heterozygosity based on the *in silico* comparison of the two CL-Brener haplotypes. Notwithstanding this, the overall analysis presented in this paper is clearly focused on a core of genes that are single copy. Moreover, because of the way in which the bioinformatic strategy was devised, we are probably underestimating the number of highly polymorphic genes. Theoretically, single copy genes with highly divergent alleles could end up in separate clusters, and therefore in different alignments (or as singleton sequences), due to their high number of nucleotide differences. Our bioinformatics strategy, using a second BLAST stage to detect these cases worked well overall, but a number of such cases did escape the initial automated analysis, and have been corrected by manual merging of alignments. Therefore, we cannot rule out the possibility of missing additional cases.

### Polymorphisms or fixed differences?

 When comparing nucleotide changes between different species, it is mostly correct to assume that these differences are *fixed* (i.e. they are substitutions or mutations). In our particular case, we are analyzing changes within a species. Therefore we have chosen to call all observed nucleotide changes as *polymorphisms* or *SNPs*. These distinctions affect the choice of different estimates of selection, such as *dN* (the number of non-synonymous changes per non-synonymous site) and *dS* (the number of synonymous changes per synonymous site), or the nucleotide diversity (π) [46]. In this work we have decided to include results based on the use of the nucleotide diversity (π) as an estimate of selection instead of the dN/dS ratio. However, it should be noted that published results [21], and our own data from an ongoing re-sequencing study focussed on selected *loci*, show that a significant percentage of the identified changes can be verified by re-sequencing a different strain from the same evolutionary lineage. This percentage can be as high as ~ 96% for changes identified between the CL-Brener haplotypes (verified in strains from the corresponding parental lineages (R. Cosentino, L. Panunzi, F. Agüero, unpublished results), strongly suggesting that the great majority of the polymorphisms identified in this study are actually *fixed differences.* This is also in agreement with recent comparisons of two *T. brucei* genomes [61]. Nonetheless, the conclusions of analyses performed in this work (see Figures 2, 3, 4, and 5), obtained using the *π* indicator, do not change subtantially if we use the dN/dS ratio instead (data not shown).

### SNP types, functional changes

When we analyzed the set of high-quality SNPs identified according to the type of change they introduce at the protein level, we observed a slightly higher number of non-synonymous SNPs. This result is in agreement with similar observations made in other protozoan organisms: i) the comparison of the genomes of *T. brucei brucei* and *T. brucei gambiense*[61]; and ii) SNPs between *Toxoplasma gondii* strains from the 3 main evolutionary lineages available at ToxoDB [62]. However, this is in contrast to observations in other eukaryotes, such as humans and helminths (*Schistosoma mansoni*[63]) where a higher number of synonymous changes is observed. This observation most likely reflects the accumulation of changes in *T. cruzi* and the other protozoan genomes through a large time of divergence. The fact that most of the allelic diversity observed within the CL-Brener hybrid has been acquired through the proposed hybridization events between ancestral individuals from lineages TcII and TcIII, imply that the observed genetic diversity within the CL-Brener clone corresponds to the expected diversity between *T. cruzi* DTUs TcII and TcIII. As reported recently in an analysis of 32 *loci* in an expanded panel of strains, the distances between the Esmeraldo-like (TcII) and non-Esmeraldo-like (TcIII) alleles of CL-Brener were the maximum observed within *T. cruzi*[21]. The estimates for time of divergence for these ancestral lineages, therefore, are in agreement with a large time of divergence, justifying the observed number of synonymous and non-synonymous sites.

The distribution of synonymous SNPs along the coding regions was uniform. However in the case of non-synonymous SNPs, we observed a tendency for accumulation in signal-peptides, *trans-*membrane domains, and cleaved C-terminal tails (C-termini that are cleaved after the addition of glycosylphosphatidyl inositol anchors). Although not statistically significant at the genome scale, we did find a number of cases in which there was striking contrast in the accumulation of non-synonymous changes between natively unstructured vs globular regions.

A similar accumulation of non-synonymous changes in these regions has been reported by others in different biological models. In yeast, a survey of SNP data from 64 strains of *Saccharomyces cerevisiae* suggest that this accumulation is particularly associated with proteins whose function is related to adaptive processes [64]. Although *T. cruzi* lags behind *S. cerevisiae* in functional characterization of its protein coding gene set, it is tempting to speculate that a similar trend is likely to be observed. Proteins carrying natively unstructured or disordered domains, such as the human transcriptional regulator p53, are often at the center of large signaling networks, and serve as organizing scaffolds for protein complexes [65,66]. In our dataset, a number of such proteins can be readily identified (see Results). One of these is the A2rel protein first described in *Leishmania donovani*[43,44]. This is a protein of unknown function, that is expressed in the amastigote stages infecting host cells, and that has been implied in the process of visceralization of infection, as it is absent in *Leishmania* species causing cutaneous leishmaniasis. Recently, this protein has been shown to be upregulated during a temperature shift similar to that experienced by the parasite upon transmission from the insect to the vertebrate host (see Table 3 in [67]). It would be interesting to analyze the function of this protein in *T. cruzi* and also the functional consequences of the observed allelic diversity.

As reviewed recently by Uversky and colleagues [65], intrinsically disordered proteins are particularly attractive targets for modulating protein-protein interactions. However, in light of the observed allelic diversity in these domains, it would be important to take this into consideration when assaying these proteins.

### Estimates of *T. cruzi* genetic diversity

The genetic diversity identified in this work gives a broad overview of the diversity present within the *T. cruzi* species, at a genomic scale. Based on the genome data analyzed herein, and on the limited re-sequencing of selected *loci* in a panel of representative strains (minimum of 3 per DTU [31], as well as other unpublished data, including the draft genomes of TcII and TcI strains), we estimate that ~ 50-60% of the genetic diversity of the species has been sampled. This is of course a gross estimate, but the support for this figure comes from the following observations. First, in our re-sequencing experiments we have found that 42% of the nucleotide changes can be discovered just by analyzing the CL-Brener hybrid. This is in agreement with other studies using a panel of reference strains [21]. This diversity corresponds to that observed between extant TcII and TcIII strains, and appears to be the maximal divergence observed between evolutionary lineages [21]. Sencondly, when re-sequencing the same *loci* in TcI strains, it was possible to identify new additional SNPs, corresponding to ~ 18% of the total genetic diversity that can be uncovered in this panel of 18 strains. However, 82% of the observed SNPs had already been identified by analyzing the CL-Brener hybrid genome. A similar figure (~17% new SNPs) is obtained for TcIV strains. However, by sequencing TcII or TcIII strains, only a minimal number of additional SNPs are uncovered, corresponding to 3-4% of the total. This reflects the fact that the majority of these nucleotide changes have already been identified from CL-Brener alleles. The same low rate of discovery of new SNPs is observed when re-sequencing strains from the TcV lineage. Strains from this lineage show very limited genetic diversity when compared with the TcVI strains. Although these observations are based on a small scale re-sequencing study, the same trend can be observed when analyzing additional *loci* from the draft genomic data for TcI and TcII strains (data not shown). According to this analysis, the next step to significantly increase the coverage of the genetic diversity identified for *T. cruzi*, should be the analysis of complete genomic or transcriptomic data from a TcIV strain.

## Conclusions

By taking advantage of the genomic and transcriptomic sequence data from a number of strains representative of different evolutionary lineages of *T. cruzi*, we have compiled an initial map of genetic diversity for this important parasite, focused mostly on protein coding, single-copy regions of the genome. The picture emerging from this analysis reflects the highly divergent nature of the ancestral haplotypes of the hybrid CL-Brener strain. However, the analysis also shows that there is a highly conserved core of the genome under apparent purifying selection, and highlights a number of genes and domains deviating from this extreme. The work represents the first genome-wide map of genetic diversity for *T. cruzi*, covering about half of the estimated nucleotide diversity of the species.

## Methods

### Data sources

Data used for SNP identification included: the *T. cruzi* CL-Brener (DTU/genotype TcVI) and Sylvio X10 (DTU/genotype TcI) genomes (coding sequences, contigs, scaffolds; obtained from GenBank using the accession numbers AAHK00000000 [24], and ADWP00000000 [28], respectively), RNA-seq (transcriptomic) data from the TcAdriana strain (DTU TcI, obtained by 454 high-throughput sequencing of an epimastigote cDNA library, Westeergard G and Vazquez MP, manuscript in preparation), partial shotgun (genomic) data from the Esmeraldo strain (DTU TcII, obtained from the NCBI Sequence Read Archive), as well as other *T. cruzi* sequences obtained from GenBank in May, 2007 (see Table 1). *T. cruzi* ESTs were obtained from dbEST and manually curated to extract information about their source (strain) [68]. To validate SNPs identified in a limited number of genes, we have checked preliminary assemblies from the JRcl4 (DTU TcI), and Esmeraldo cl3 (DTU TcII) strains available at the TriTrypDB resource [39].

### Sequence clustering and alignment

Before clustering, sequences were screened against a library of *T. cruzi* repetitive elements and vector (plasmid) sequences and the corresponding matching regions were masked [33]. Sequences containing at least 50 bp of non-masked bases were further processed. *T. cruzi* coding sequences from the genome project (CDS), and other nucleotide (mRNA, EST, genomic DNA) sequences were mapped to the reference genome sequence scaffolds using BLAT [69]. Sequences were grouped in clusters based on the following stringent mapping: any two sequences overlapping in their mapping against a given reference genome scaffold were put together into the same cluster. Subsequently, multiple sequence alignments (MSAs) were obtained for each cluster using phrap (http://www.phrap.org). When more than one multiple sequence alignment was obtained for a group of clustered sequences, we manually merged the alignments if they met the following criteria: i) the sequences from the reference genome did not have an assembly warning note; ii) there were no more than two reference coding sequences from the CL-Brener genome in the merged alignment; and iii) the divergence between the reference sequences, as judged by manual inspection, would justify the merging (for example, manual inspection of these cases allowed us to merge cases of reference sequences differing only in the extension of their 5’ ends – usually arising from different annotation of the translational start codon in each allele – or in indels larger than a couple of codons, which would otherwise cause the sequences to end up in separate contigs due to the stringent criteria used in this step). Genomic data from the Sylvio X10 draft assembly, and from reconstructed transcripts from the TcAdriana 454 RNA-seq data were mapped against these CL-Brener reference alignments. Finally, the quality of each MSA was assessed using two parameters: the % identity, and an estimate of the effective length of the alignment. This latter figure provides the percentage of columns in the alignment where the alignment depth >= 2. MSAs with a % identity > 95% were considered further. MSAs that did not meet these criteria were manually inspected to evaluate further manual editing (split alignments). Because in some cases alignments might contain sequences that are shorter at one or both ends, the effective length of the alignment (ignoring regions covered by a single sequence) was used to compute alignment attributes, such as the SNP density.

### SNP identification and scoring

Multiple sequence alignments were scanned using a custom Perl script to identify polymorphic columns. These putative SNPs were analyzed using PolyBayes [34] to obtain a numeric score for each SNP (the probability of this being a true polymorphism). PolyBayes can use individual base quality values for the calculation of SNP probabilities, but because original chromatogram data is not available for all sequences, we have assigned arbitrary base quality values to these sequences. These arbitrary quality values were derived based on the estimated sequencing error rate for the different types of sequences used: EST sequences were assigned an arbitrary base quality value of 20 (1 error per 100 bases), which is typical for single-pass, unedited sequences; while sequences derived from individual submissions to GenBank were assigned a quality value of 30 (1 error per 1000 bases). Sequences derived from the genome project were assigned a base quality value of 40 (1 error per 10,000 bases), based on the fact that they are derived from an ~11X coverage of the genome.

The nucleotide diversity indicator π (average proportion of nucleotide differences between all possible pairs of sequences) [45] was calculated for each alignment using the effective length of the multiple sequence alignment (not considering regions of the alignment where the depth was 1) as the total number of available sites.

### SNP validation by re-sequencing

DNA from the CL-Brener and Sylvio X10 strains of *T. cruzi* was prepared by extraction with DNAZol (Invitrogen), according to the protocol provided by the supplier. To select the regions for amplification, we started from a multiple sequence alignment containing putative polymorphic sites, and identified regions in the alignment that correspond to blocks of conserved sequence (100% identity, devoid of indels and putative SNPs). Two such regions separated by a block of 400–600 bp and containing at least 2 putative polymorphic sites were selected in order to design the corresponding 5’ and 3’ amplification primers. The genes and oligonucleotides used for PCR-based re-sequencing are listed in the Additional file 10: Table S7. These oligonucleotide primers were used both for the amplification and sequencing reactions. Each 25 μl reaction was composed of 2.5 U of Taq polymerase (Invitrogen), 2.5 μl 10X Buffer (Invitrogen), 2 μl 2.5 mM dNTPs (Invitrogen), 0.8 μl 50 mM MgCl_2_ (Invitrogen) and 16.2 μl MiliQ water (Millipore). Amplification products were checked in 1.2% agarose gels stained with ethidium bromide and if a single amplification product was observed, an aliquot of the amplification reaction was treated with Exonuclease I (Fermentas) and Shrimp Alkaline Phosphatase (SAP) (Fermentas) and two sequencing reactions were prepared, each with one of the primers used for the amplification of the product. Sequencing was carried out in an Applied Biosystems 3130 capillary sequencer using a Big-Dye terminator cycle sequencing kit, according to the instructions of the manufacturer. Chromatogram data derived from these reactions was analyzed using PolyPhred [35] to identify polymorphisms, including heterozygous peaks. Information was collected for both homozygous high quality discrepancies between sequences and heterozygous peaks within sequences.

### Target prioritization strategy

To prioritize targets, we used the functionality available within the TDR Targets database to assign different scores to genes depending on the presence/absence of different attributes or features [54,70]. Briefly, in TDR Targets (http://tdrtargets.org) we ran a query for each selected attribute to filter the genome and obtain a subset of genes (for example, those with orthologs in other kinetoplastids). After running all queries we assigned different numerical weights to each subset of genes (see below), and calculated a weighted union of these lists. The final *score* of a gene is the cumulative sum of the weights derived from each list in which the gene was present. This possibility of obtaining weighted unions (instead of intersections) allowed us to make a comprehensive prioritization of the *T. cruzi* genome using a large number of criteria. The following attributes were evaluated (the score/penalty applied to each criteria is available in Additional file 9: Table S6): essentiality of bacterial (*E. coli*, *M. tuberculosis*), yeast (*S. cerevisiae*) or *C. elegans*) orthologs; absence of orthologs in mammals (human/mouse); presence of orthologs in other kinetoplastids (*T. brucei*, *L. major*); proteomic evidence of expression in amastigotes and trypomastigotes (data obtained form TriTrypDB [39]); availability of literature records for the target; availability of biochemical assays for the target; precedence for production of soluble recombinant protein; low molecular weight (<100 kDa); target is an enzyme (has an EC number); target has a solved 3D structure or a calculated 3D model; target has *trans*-membrane spanning domains (penalized); target is present in multiple copies (> 2 copies per haploid genome) (penalized); presence of a signal peptide (ER-secretory route) (penalized); presence of a GPI signal sequence (penalized). To further improve on this prioritization, we assigned positive scores to genes that are likely to be under purifying selection (low π values). The score for each gene was calculated using a sigmoid normalization function:

$$\mathrm{score}\left(\pi ,\;k\right)=1+20^{-\pi /k}*50$$

where π is the nucleotide diversity and *k* is a scaling factor (0.08 in this case). A list of genes with their corresponding scores calculated in this way was uploaded to TDR Targets as a tab-delimited text file, to finish the prioritization strategy. The result of this prioritization is shown in the Additional file 9: Table S6 and is also available from TDR Targets (http://tdrtargets.org/published/browse/423).

### Locus identifiers

According to recent changes related to standardization in trypanosomatid locus identifiers used in community databases, all such *T. cruzi* identifiers referenced in this work appear in their current shorter form, e.g. TcCLB.507853.10 (equivalent to the old locus identifier Tc00.1047053507853.10).

### Data deposition

The sequences reported in this paper have been deposited in the GenBank database (accession numbers: HQ586966-HQ587039; SNPs identified in this paper have been deposited in the dbSNP database (submission batch id: 1050246) and are also available from the TcSNP database at http://snps.tcruzi.org. All TcSNP alignment identifiers mentioned in this work (e.g. tcsnp:438249) can be accessed using the following standard URI, and replacing the alignment identifier (e.g. 438249) in each case: http://snps.tcruzi.org/genes/alignment?assembly_id=438249.

## Abbreviations

SNP: Single nucleotide polymorphism; DTU: Discrete typing unit.

## Competing interests

The authors declare that no competing interests exist.

## Authors' contributions

AA and LP assembled the alignments of CL-Brener coding sequences, LP mapped coding sequences from draft genome assemblies, AA and LP identified and characterized SNPs, performed statistical analysis, and drafted the manuscript. DOS participated in the design of the study. FA conceived the study, participated in its design and coordination, and wrote the manuscript. LP and ROC carried out the re-sequencing study to validate SNPs. All authors read and approved the final manuscript.

## Supplementary Material

Additional file 1 **Table S1.** Complete list of alignments used for the detection of SNPs in this work. The table contains information on the alignments and the type and number of SNPs identified in each case. The Calc/Excel workbook contains two spreadhseets: the first contains a complete legend, describing all columns in detail; the second contains the data itself. Click here for file

Additional file 2 **Table S2.** Experimental validation of identified SNPs by PCR-based re-sequencing. The table shows, for each *loci* analyzed, the number of SNPs identified *in silico*, as well as those that have been validated (V) or not (NV) by re-sequencing. Click here for file

Additional file 3 **Table S3.** List of nonsense polymorphisms identified in this work. For each nonsense SNP identified we show the corresponding codon for each genome/strain analyzed, and N/A if the gene has not been identified in a dataset. Click here for file

Additional file 4 **Figure S1.** Nonsense SNPs observed in the CL-Brener genome. **(A)** List of observed nonsense SNPs, sorted by descending frequency. Nonsense changes shown in black require only a single mutation to produce a stop codon. SNPs shown in color require changing two bases in the same codon (Ts = transition; Tv = transversion). **(B)** List of observed nonsense mutations, by type (Ts/Tv). **(C)** Genetic code showing all possible single base changes that would produce a stop codon, and those observed in T. cruzi. Click here for file

Additional file 5 **Figure S2.** SNP density in globular vs unstructured protein domains. The density of SNPs in globular vs intrinsically unstructured regions (predicted by IUPred) was compared for synonymous, and non-synonymous changes (left and middle panels). A third panel (right) showing all SNPs is shown for comparison. In all cases the differences between the distribution were not statistically different. Click here for file

Additional file 6 **Figure S3.** A natively unstructured domain accumulating non-synonymous changes (complete alignment). Multiple sequence alignment showing the TcCLB.506553.20 gene, and its allelic counterparts in other strains (Additional file 10: Figure S3). The gene has a globular N-terminal domain, and an intrinsically unstructured C-terminal domain, as predicted by IUPred [73]. This figure contains the complete alignment shown in Figure 1. Click here for file

Additional file 7 **Table S4.** GO Slim annotation of alignments used for the detection of SNPs in this work. Click here for file

Additional file 8 **Table S5.** KEGG pathway mapping of alignments used for the detection of SNPs in this work. Click here for file

Additional file 9 **Table S6.** Drug target prioritization using a score based on the nucleotide diversity estimator (π) as one of the main driving criteria. The spreadhsheet contains the top 300 targets. The complete ranked proteome, as well as the individual queries can be accessed online at http://tdrtargets.org/published/browse/t/423. Click here for file

Additional file 10 **Table S7.** Primers and amplification products used in the SNP validation experiment. Click here for file
