# Supplementary material for: A genomic scale map of genetic diversity in Trypanosoma cruzi
Source: BMC Genomics. 2012 Dec 27;13:736. doi: 10.1186/1471-2164-13-736 (PMC3545726; doi:10.1186/1471-2164-13-736)
Supplement: Additional file 4 — Figure S1. Nonsense SNPs observed in the CL-Brener genome. (A) List of observed nonsense SNPs, sorted by descending frequency. Nonsense changes shown in black require only a single mutation to produce a stop codon. SNPs shown in color require changing two bases in the same codon (Ts = transition; Tv = transversion). (B) List of observed nonsense mutations, by type (Ts/Tv). (C) Genetic code showing all possible single base changes that would produce a stop codon, and those observed in T. cruzi. [file 1471-2164-13-736-S4.pdf]

A

Nonsense mutations	Type	Codon pos	Frequency
CAG → TAG	*Ts*	1	15
CAA → TAA	*Ts*	1	9
CGA → TGA	*Ts*	1	6
GAA → TAA	*Tv*	1	6
TGG → TGA	*Ts*	3	6
TAC → TAA	*Tv*	3	5
TGG → TAG	*Ts*	2	5
TCA → TAA	*Tv*	2	4
AAA → TAA	*Tv*	1	3
GAG → TAG	*Tv*	1	3
GGA → TGA	*Tv*	1	3
TAT → TAG	*Tv*	3	2
TTA → TAA	*Tv*	2	2
AAG → TAG	*Tv*	1	1
AGG → TGA	*Ts/Tv*	***1, 3***	***1***
CAT → TAG	*Ts/Tv*	***1, 3***	***1***
CGG → TAG	*Ts/Ts*	***1, 2***	***1***
GGG → TAG	*Ts/Tv*	***1, 2***	***1***
TAT → TAA	*Tv*	3	1
TAT → TGA	*Ts/Tv*	***2, 3***	***1***
TCA → TGA	*Tv*	2	1
TCG → TAG	*Tv*	2	1
TGC → TGA	*Tv*	3	1

Observed nonsense mutations (2 SNPs required)

B

Change	Type	Ocurrences
A ↔ G	*Ts*	16
C ↔ T	*Ts*	32
C ↔ G	*Tv*	1
A ↔ C	*Tv*	10
G ↔ T	*Tv*	12
A ↔ T	*Tv*	13

C

	Codon	Mut
A	***AAA***	*TAA*
	AAC	
	AAG	*TAG*
	AAT	
	ACA	
	ACC	
	ACG	
	ACT	
	AGA	TGA
	AGC	
	AGG	
	AGT	
	ATA	
	ATC	
	ATG	
	ATT	

	Codon	Mut
C	***CAA***	*TAA*
	CAC	
	CAG	*TAG*
	CAT	
	CCA	
	CCC	
	CCG	
	CCT	
	CGA	*TGA*
	CGC	
	CGG	
	CGT	
	CTA	
	CTC	
	CTG	
	CTT	

	Codon	Mut
G	***GAA***	*TAA*
	GAC	
	GAG	*TAG*
	GAT	
	GCA	
	GCC	
	GCG	
	GCT	
	GGA	*TGA*
	GGC	
	GGG	
	GGT	
	GTA	
	GTC	
	GTG	
	GTT	

	Codon	Mut	Mut
T	TAA		
	TAC	*TAA*	TAG
	TAG		
	TAT	*TAA*	*TAG*
	TCA	*TAA*	*TGA*
	TCC		
	TCG	*TAG*	
	TCT		
	TGA		
	TGC	*TGA*	
	TGG	*TAG*	*TGA*
	TGT	TGA	
	TTA	*TAA*	TGA
	TTC		
	TTG	TAG	
	TTT		

Canonical stop codons
Observed nonsense mutations (1 SNP required)
Unobserved nonsense mutations (1 SNP required)

Nonsense mutations observed in the CL-Brener genome. (A) List of observed nonsense mutations, sorted by descending frequency. Nonsense mutations shown in black require only a single mutation to produce a stop codon. Mutations shown in color require changing two bases in the same codon (Ts = transition; Tv = transversion). **(B)** List of observed nonsense mutations, by type (Ts/Tv). **(C)** Genetic code showing all possible single base changes that would produce a stop codon, and those observed in *T. cruzi*.
