# Supplementary figures and images for: A genomic scale map of genetic diversity in Trypanosoma cruzi
Source: BMC Genomics. 2012 Dec 27;13:736. doi: 10.1186/1471-2164-13-736 (PMC3545726; doi:10.1186/1471-2164-13-736)

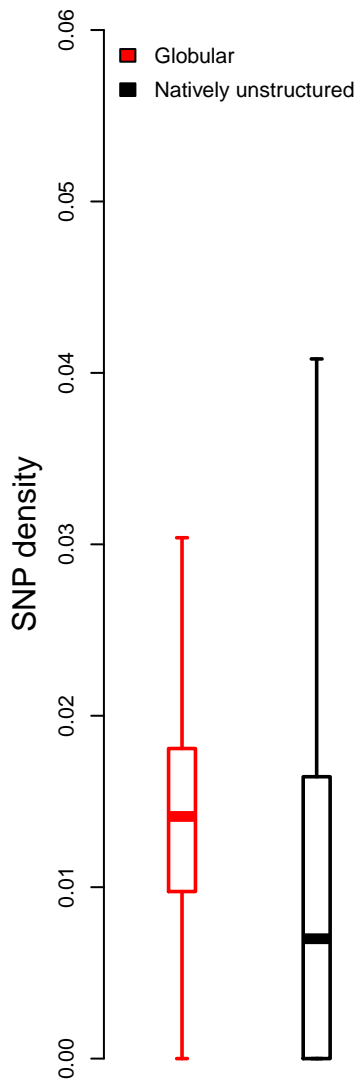

Syn SNPs

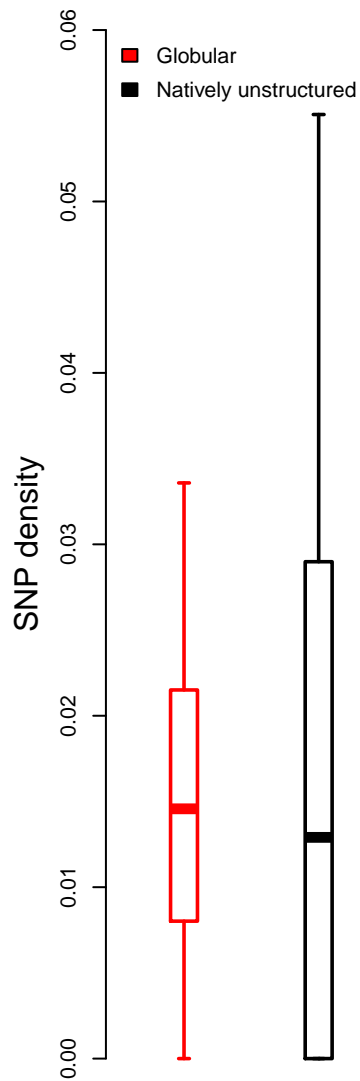

Non-Syn SNPs

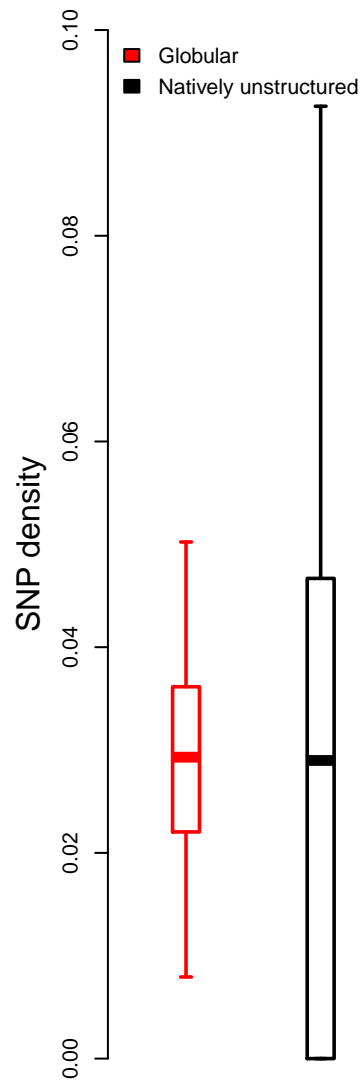

All SNPs

Supplement: Additional file 5 — Figure S2. SNP density in globular vs unstructured protein domains. The density of SNPs in globular vs intrinsically unstructured regions (predicted by IUPred) was compared for synonymous, and non-synonymous changes (left and middle panels). A third panel (right) showing all SNPs is shown for comparison. In all cases the differences between the distribution were not statistically different. [file 1471-2164-13-736-S5.pdf]
