# Supplementary material for: A genomic scale map of genetic diversity in Trypanosoma cruzi
Source: BMC Genomics. 2012 Dec 27;13:736. doi: 10.1186/1471-2164-13-736 (PMC3545726; doi:10.1186/1471-2164-13-736)
Supplement: Additional file 6 — Figure S3. A natively unstructured domain accumulating non-synonymous changes (complete alignment). Multiple sequence alignment showing the TcCLB.506553.20 gene, and its allelic counterparts in other strains (Additional file 10: Figure S3). The gene has a globular N-terminal domain, and an intrinsically unstructured C-terminal domain, as predicted by IUPred [73]. This figure contains the complete alignment shown in Figure 1. [file 1471-2164-13-736-S6.pdf]

1. Esmeraldo cl3
2. TcCLB.506553.20
3. TcCLB.507769.30
4. JR cl4
5. TcI
6. Sylvio X10

Amino Acid Color Code

Charged
- D E — Acidic
- K R H — Basic

Hydrophobic
- F Y W — Aromatic
- I L V — Aliphatic

Polar, neutral
- C M — -SH containing
- S T — -OH containing
- N Q — $-NH_2$ containing

Small
- G A

Unique
- P

						16						34						52					
1	ATG	GGG	AAG	CGT	GGG	CGT	GAG	GGG	ACT	TTG	GAC	AAG	GCG	CGC	TGC	CTT	GCT	TTT	GTG	CAT	CAG	CTT	TGG
2	ATG	GGG	AAG	CGT	GGG	CGT	GAG	GGG	ACT	TTG	GAC	AAG	GCG	CGC	TGC	CTT	GCT	TTT	GTG	CAT	CAG	CTT	TGG
3	ATG	GGG	AAG	CGT	GGG	CGT	GAG	GGG	ACT	TTG	GAC	AAG	CCG	CGC	TGC	CTT	GCT	TTT	GTA	CAT	CAG	CTT	TGG
4	ATG	GGG	AAG	CGT	GGG	CGT	GAG	GGG	ACT	TTG	GAC	AAG	GCG	CGC	TGC	CTT	GCT	TTT	GTG	CAT	CAG	CTT	TGG
5	ATG	GGG	AAG	CGT	GGG	CGT	GAG	GGG	ACT	TTG	GAC	AAG	GCG	CGC	TGC	CTT	GCT	TTT	GTG	CAT	CAG	CTT	TGG
6	ATG	GGG	AAG	CGT	GGG	CGT	GAG	GGG	ACT	TTG	GAC	AAG	GCG	CGC	TGC	CTT	GCT	TTT	GTG	CAT	CAG	CTT	TGG

	70						88						106						124				
1	GAC	AAG	GAC	AAG	CTC	AAG	ATG	TTT	CAC	CAC	CCA	GTC	AGC	GCT	GCG	GAG	CTG	CCG	GAC	TAC	CAC	AAG	GTC
2	GAC	AAG	GAC	AAG	CTC	AAG	ATG	TTT	CAC	CAC	CCA	GTC	AGC	GCT	GCG	GAG	CTG	CCG	GAC	TAC	CAC	AAG	GCC
3	GAC	AAG	GAC	AAG	CTC	AAG	ATG	TTT	CAC	CAC	CCA	ATC	AGC	GCT	GCG	GAG	CTG	CCG	GAC	TAC	CAC	AAG	GTC
4	GAT	AAG	GAC	AAG	CTC	AAG	ATG	TTT	CAC	CAC	CCA	ATC	AGC	GCT	GCG	GAG	CTG	CCG	GAC	TAC	CAC	AAG	GTC
5	GAT	AAG	GAC	AAG	CTC	AAG	ATG	TTT	CAC	CAC	CCA	ATC	AGC	GCT	ACG	GAG	CTG	CCG	GAC	TAC	CAC	AAG	GTC
6	GAT	AAG	GAC	AAG	CTC	AAG	ATG	TTT	CAC	CAC	CCA	ATC	AGC	GCT	GCG	GAG	CTG	CCG	GAC	TAC	CAC	AAG	GTC

		142						160						178						196			
1	GTA	AAT	TAT	CCC	GTT	GAC	CTG	TCG	ACA	ATT	CGC	CAG	GGC	ATT	GAG	TCC	GGA	ACG	TAC	GAC	AGC	GAT	GCG
2	ATA	AAT	TAT	CCC	GTT	GAC	CTG	TCG	ACA	ATT	CGC	CAG	GGC	ATT	GAG	TCC	GGA	ACG	TAC	GAC	AGC	GAT	GCG
3	ATA	AAT	TAT	CCC	GTT	GAC	CTG	TCG	ACA	ATT	CGT	CAG	GGC	ATT	GAG	TCC	GGA	AAG	TAC	GAC	AGC	GAT	GCG
4	ATA	AAT	TAT	CCT	GTT	GAC	CTG	TCG	ACA	ATT	CGT	CAG	GGC	ATT	GAG	TCC	GGA	AAG	TAC	GAC	AGC	GAT	GCG
5	ATA	AAT	TAT	CCT	GTT	GAC	CTG	TCG	ACA	ATT	CGT	CAG	GGC	ATT	GAG	TCC	GGA	AAG	TAC	GAC	AGC	GAT	GCG
6	ATA	AAT	TAT	CCT	GTT	GAC	CTG	TCG	ACA	ATT	CGT	CAG	GGC	ATT	GAG	TCC	GGA	AAG	TAC	GAC	AGC	GAT	GCG

			214						232						250						268		
1	GAT	GTG	CAG	AAC	GCC	GTT	GCG	CAG	ATG	ATT	GCC	AAC	GCA	CTG	GAG	TAC	AAC	GCA	AAG	GGG	ACT	GAG	TGG
2	GAT	GTG	CAG	AAC	GCC	GTT	GCG	CAG	ATG	ATT	GCC	AAC	GCA	CTG	GAG	TAC	AAC	GCA	AAG	GGG	ACT	GAG	TGG
3	GAT	GTG	CAG	AAC	GCC	GTT	GCG	CAG	ATG	ATT	GCC	AAC	GCA	CTG	GAG	TAC	AAC	GCA	AAG	GGG	ACT	GAG	TGG
4	GAT	GTG	CAG	AAC	GCC	GTT	GCG	CAG	ATG	ATT	GCC	AAC	GCA	CTG	GAG	TAC	AAC	GCA	AAG	GGG	ACT	GAG	TGG
5	GAT	GTG	CAG	AAC	GCC	GTT	GCG	CAG	ATG	ATT	GCC	AAC	GCA	CTG	GAG	TAC	AAC	GCA	AAG	GGG	ACT	GAG	TGG
6	GAT	GTG	CAG	AAC	GCC	GTT	GCG	CAG	ATG	ATT	GCC	AAC	GCA	CTG	GAG	TAC	AAC	GCA	AAG	GGG	ACT	GAG	TGG

				286						304						322						340	
1	CAC	CAT	CAG	GCG	CTG	TCG	TTC	CGA	AAC	ATT	TAC	TTG	GAC	GTT	GCA	AGG	CAA	TGC	GGG	CTG	AGT	GTT	GAC
2	CAC	CAT	CAG	GCG	CTG	TCG	TTC	CGA	AAC	ATT	TAC	TTG	GAC	GTT	GCA	AGG	CAA	TGC	GGG	CTG	AGT	GTT	GAC
3	CAC	CAA	CAG	GCG	CTG	TCG	TTC	CGA	AAC	ATT	TAC	TTG	GAC	GTT	GCA	AGG	CAA	TGC	GGG	CTG	AGT	GTT	GAC
4	CAC	CAA	CAG	GCG	CTG	TCG	TTC	CGA	AGC	ATT	TAC	TTG	GAC	GTT	GCA	AGG	CAA	TGC	GGG	CTG	AGT	GTT	GAC
5	CAC	CAA	CAG	GCG	CTG	TCG	TTC	CGA	AGC	ATT	TAC	TTG	GAC	GTT	GCA	AGG	CAA	TGC	GGG	CTG	AGT	GTT	GAC
6	CAC	CAA	CAG	GCG	CTG	TCG	TTC	CGA	AGC	ATT	TAC	TTG	GAC	GTT	GCA	AGG	CAA	TGC	GGG	CTG	AGT	GTT	GAC

					358						376						394						412
1	GAC	GAC	GCG	GCG	TAC	ATC	CCA	TCG	GCG	GCT	TTC	AAG	GAC	AAT	GAG	TCG	ACT	CTG	CGC	AAG	GCG	GAG	AAG
2	GAC	GAC	GCG	GCG	TAC	ATC	CCA	TCG	GCG	GCT	TTC	AAG	GAC	AAT	GAG	TCG	ACT	CTG	CGC	AAG	GCG	GAG	AAG
3	GAC	GAC	GCG	GCG	TAC	ATC	CCA	TCG	GCG	GCT	TTC	AAG	GAC	GAT	GAG	TCG	ACT	CTA	CGC	AAG	GCG	GAG	AAG
4	GAC	GAC	GCG	GCG	TAC	ATC	CCA	TCG	GCG	GCT	TTC	AAG	GAC	GAT	GAG	TCG	ACT	CTA	CGC	AAG	GCG	GAG	AAG
5	GAC	GAC	GCG	GCG	TAC	ATC	CCA	TCG	GCG	GCT	TTC	AAG	GAC	GAT	GAG	TCG	ACT	CTA	CGC	AAG	GCG	GAG	AAG
6	GAC	GAC	GCG	GCG	TAC	ATC	CCA	TCG	GCG	GCT	TTC	AAG	GAC	GAT	GAG	TCG	ACT	CTA	CGC	AAG	GCG	GAG	AAG

						430						448						466					
1	AAA	ATT	GCG	GAG	AAT	CTC	GAC	GTG	GTA	CTG	AAG	GAC	CTC	GAG	GAG	GAG	AAG	GCG	GTG	CCA	CTG	GAG	GAG
2	AAA	ATT	GCG	GAG	AAT	CTC	GAC	GTG	GTA	CTG	AAG	GAC	CTC	GAG	GAG	GAG	AAG	GCG	GTG	CCA	CTG	GAG	GAG
3	AAA	ATT	GCG	GAG	AAT	CTC	GAC	GTG	GTA	CTG	AAG	GAC	CTC	GAG	GAG	GAA	AAG	GCG	GTG	CCG	CTG	GAG	GAG
4	AAA	ATT	GCG	GAA	AAT	CTC	GAC	GTG	GTA	CTG	AAG	GAC	CTC	GAG	GAG	GAC	AAG	GCG	GTG	CCG	CTG	GAG	GAG
5	AAA	ATT	GCG	GAA	AAT	CTC	GAC	GTG	GTA	CTG	AAG	GAC	CTC	GAG	GAG	GAC	AAG	GCG	GTG	CCG	CTG	GAG	GAG
6	AAA	ATT	GCG	GAA	AAT	CTC	GAC	GTG	GTA	CTG	AAG	GAC	CTC	GAG	GAG	GAC	AAG	GCG	GTG	CCG	CTG	GAG	GAG

	484						502						520						538				
1	CTT	AGA	GCG	AAG	TAC	AAG	CGT	GCG	GAG	AAG	GAG	ACA	GGA	AGC	CAG	AAT	GCG	GAT	GGG	AGT	GAT	GGC	AAC
2	CTT	AGA	GCG	AAG	TAC	AAG	CGT	GCG	GAG	AAG	GAG	ACA	GGA	AGC	CAG	AAT	GCG	GAT	GGG	AGT	GAT	GGC	AAC
3	CTT	AAA	GCG	AAG	TAC	AAG	CGT	GCG	AAA	AAG	AAG	AAA	GGA	GGC	AAG	AAT	GCG	GAT	GGG	AGT	GAC	AGC	GAT
4	CTT	AGA	GCG	AAG	TAC	AAG	CAT	GCG	GAA	AAG	AAG	AAA	GGA	GGC	AAG	AAT	GCG	GAT	GTG	AGT	GAC	AGC	---
5	CTT	AGA	GCG	AAG	TAC	AAG	CGT	GCG	GAA	AAG	AAG	AAA	GGA	GGC	AAG	AAT	GCG	GAT	GTG	AGT	GAC	AGC	---
6	CTT	AGA	GCG	AAG	TAC	AAG	CGT	GCG	GAA	AAG	AAG	AAA	GGA	GGC	AAG	AAT	GCG	GAT	GTG	AGT	GAC	AGC	---

		556						574						592						610			
1	AGT	GAT	GAC	ACT	GAA	GAT	GAG	GAA	TTT	GAC	AGC	GAT	GAT	AGC	CGT	GAG	AGC	GAT	GAC	GAC	AGC	GAT	GAT
2	AGT	GAT	GAC	ACT	GAA	GAT	GAG	GAA	TTT	GAC	AGC	GAT	GAT	AGC	CGT	GAG	AGC	GAT	GAC	GAC	AGC	GAT	GAT
3	GAC	AGC	GAT	GAC	AGC	GAT	GAC	AGC	GAT	GAG	AGC	GAA	GAG	AGC	GAA	GAG	AGC	GAT	GAG	ATC	GCT	GAT	GAC
4	---	---	---	---	---	---	---	---	GAT	GAG	AGC	GAA	GAG	AGC	GAA	GAG	AGC	GAT	GAG	ATC	GCT	GAT	GAC
5	---	---	---	---	---	GAT	GAG	AGC	GAA	GAG	AGC	GAA	GAG	AGC	GAA	GAG	AGC	GAT	GAG	ATC	GCT	GAT	GAC
6	---	---	---	---	---	GAT	GAG	AGC	GAA	GAG	AGC	GAA	GAG	AGC	GAA	GAG	AGC	GAT	GAT	GAC	AGT	GAT	---

			628						646						664						682		
1	GAT	GAT	---	---	---	---	GAC	AGC	GAT	GAT	GAG	AGC	GAT	---	---	GAT	GAC	AGT	GAA	GAT	GAT	GAT	GAT
2	GAT	GAT	---	---	---	---	GAC	AGC	GAT	GAT	GAG	AGC	GAT	---	---	GAT	GAC	AGT	GAA	GAT	GAT	GAT	---
3	AGC	GAT	GAT	---	---	---	GAC	AGC	GAT	GAT	GAC	AGC	GAT	GAC	AGC	GAT	GAT	AGC	GAT	GAA	GAG	AAG	TTT
4	AGT	---	---	---	---	---	GAT	GAC	AGT	GAT	GAC	AGC	GAT	---	---	GAT	GAT	AGC	GAT	GAA	GAA	AAG	TTT
5	AGC	TGA	TGA	CAG	CGA	TGA	GAC	AGC	GAT	GAT	GAT	AGC	GAT	---	---	GAT	GAT	AGC	GAT	GAA	GAA	AAG	TTT
6	---	---	---	---	---	---	GAC	AGC	GAT	GAT	GAC	AGC	GAT	---	---	GAT	GAT	AGC	GAT	GAA	GAA	AAG	TTT

				700						718					
1	GAT	GAT	GAT	GAC	AGT	GAT	GAT	GAT	GAT	TAG	---	---	---	---	---
2	---	GAT	GAT	GAC	AGT	GAT	GAT	GAT	GAT	TAG	---	---	---	---	---
3	TAT	TAT	GAT	GAT	GAG	AGT	GAT	GAT	GAA	TAA	---	---	---	---	---
4	TAT	TGT	GAT	GAT	GAG	AGT	GAT	GAT	GAA	TTA	ATT	TTT	TTT	TTT	TTA
5	TAT	TAT	GAT	GAT	GAG	AGT	GAT	GAT	GAA	TAA	---	---	---	---	---
6	TAT	TAT	GAT	GAT	GAG	AGT	GAT	GAT	GAA	TTA	AGT	TTT	TTT	TTT	TAA
